# Transcriptome Profiling Analysis on Whole Bodies of Microbial Challenged *Eriocheir sinensis* Larvae for Immune Gene Identification and SNP Development

**DOI:** 10.1371/journal.pone.0082156

**Published:** 2013-12-04

**Authors:** Zhaoxia Cui, Xihong Li, Yuan Liu, Chengwen Song, Min Hui, Guohui Shi, Danli Luo, Yingdong Li

**Affiliations:** 1 Key Laboratory of Experimental Marine Biology, Institute of Oceanology, Chinese Academy of Sciences, Qingdao, China; 2 University of Chinese Academy of Sciences, Beijing, China; 3 National & Local Joint Engineering Laboratory for Ecological Mariculture, Institute of Oceanology, Chinese Academy of Sciences, Qingdao, China; Beijing Institute of Microbiology and Epidemiology, China

## Abstract

To study crab immunogenetics of individuals, newly hatched *Eriocheir sinensis* larvae were stimulated with a mixture of three pathogen strains (Gram-positive bacteria *Micrococcus luteus*, Gram-negative bacteria *Vibrio alginolyticus* and fungi *Pichia pastoris*; 10^8^ cfu·mL^-1^). A total of 44,767,566 Illumina clean reads corresponding to 4.52 Gb nucleotides were generated and assembled into 100,252 unigenes (average length: 1,042 bp; range: 201-19,357 bp). 17,097 (26.09%) of 65,535 non-redundant unigenes were annotated in NCBI non-redundant protein (Nr) database. Moreover, 23,188 (35.38%) unigenes were assigned to three Gene Ontology (GO) categories, 15,071 (23.00%) to twenty-six Clusters of orthologous Groups (COG) and 8,574 (13.08%) to six Kyoto Encyclopedia of Genes and Genomes (KEGG) pathways, respectively. Numerous genes were further identified to be associated with multiple immune pathways, including Toll, immune deficiency (IMD), janus kinase (JAK)-signal transducers and activators of transcription (STAT) and mitogen-activated protein kinase (MAPK) pathways. Some of them, such as tumor necrosis factor receptor associated factor 6 (TRAF6), fibroblast growth factor (FGF), protein-tyrosine phosphatase (PTP), JNK-interacting protein 1 (JIP1), were first identified in *E. sinensis*. TRAF6 was even first discovered in crabs. Additionally, 49,555 single nucleotide polymorphisms (SNPs) were developed from over 13,309 unigenes. This is the first transcriptome report of whole bodies of *E. sinensis* larvae after immune challenge. Data generated here not only provide detail information to identify novel genes in genome reference-free *E. sinensis*, but also facilitate our understanding on host immunity and defense mechanism of the crab at whole transcriptome level.

## Introduction

Chinese mitten crab *Eriocheir sinensis*, belonging to Grapsidae family of decapod crustaceans, is a catadromous species with a lifetime about two years. The crab has one reproductive season and dies shortly after reproduction [1]. After being hatched, *E. sinensis* larvae normally experience several developmental processes, including five typical Zoea-stages (Zoea I-V) and a Megalopa-stage [2]. Culture of *E. sinensis* under facility condition has started since 1980s [3] and constitutes a prospective freshwater fishery industry. It then produces tons of crabs as common food every year in China. Also, *E. sinensis* is easily to be artificially propagated and transported over long distance, which may make the species as a model organism in aquaculture studies. However, with development of intensive culture, various diseases like tremor disease (TD) and black gill syndrome (BGS) frequently occur and seriously threaten *E. sinensis* stocks. In particular, larvae of the species suffer from diseases more often than adult crabs. High mortalities of larvae can be easily caused by infection with microorganisms of *Vibrio*, *Micrococcus* and *Fungus* [4]. It is compulsory to obtain comprehensive knowledge about immune system of the crab.

Analysis of expressed sequence tags (ESTs) from cDNA library by Sanger sequencing method is proved to be useful for gene identification and expression profiling analysis. Several EST analyses of haemocytes and hepatopancreas from healthy *E. sinensis* are performed and numerous immune related sequences are consequently obtained [3,5,6]. They supply basic data for development of functional genes and molecular markers to increase disease resistance of crabs. Some immune genes are also cloned and characterized from haemocytes (haemolymph) of *E. sinensis*, such as crustin [7], antioxidative protein [8-10], antilipopolysaccharide factor (ALF) [11-13], prophenoloxidase (proPO) [14], serine proteinase (SP) and serine proteinase homologous (SPH) [15,16]. However, due to the limitation of traditional sequencing method and the tissues used for analysis, immune information of *E. sinensis* is still scattered and inadequate.

Newly-developed high-throughput sequencing technologies, such as Roche/454, Solexa/Illumina and ABI/SOLiD, furnish the opportunity to produce large numbers of sequence data in non-model organisms [17]. They provide a convenient and high-effective solution for *de novo* assembly of genome reference-free species [18]. Roche 454 pyrosequencing is a primary approach to yield transcriptomic resources and discover important genes [19-22]. However, application of this technique can be hindered by its high cost. Comparing with 454, Illumina and SOLiD provide ultra-short reads, but they are up to 30 times less expensive and produce much more sequence reads [23,24]. In recent years, Illumina method has been widely used in transcriptome analyses of various species [23,25-29]. Studies on transcriptomes from whole bodies of larvae are performed in different invertebrates, such as *Litopenaeus vannamei* [28], *Musca domestica* [30], *Galleria mellonella* [31] and *Apis cerana cerana* [32]. These reports establish fundamental data to develop extensive genomic and transcriptomic resources for invertebrate larvae.

Previously, we have identified numerous immune-related genes from transcriptome of microbial challenged *E. sinensis* hepatopancreas [33]. It provides a basis for functional classification and gene characterization of mitten crab. In the present study, whole bodies of *E. sinensis* larvae were challenged by a mixture of three pathogen strains (Gram-positive bacteria *Micrococcus luteus*, Gram-negative bacteria *Vibrio alginolyticus* and fungi *Pichia pastoris*). These pathogens represented three different types of major microbes that infected the crab and brought about serious diseases in aquaculture. The experimental analysis was expected to completely reveal sequence information, especially the important immune genes of challenged *E. sinensis*, which could be valuable to study crab immunogenetics and enhance crab resistance to various microorganisms. Besides, SNPs that were ready for marker development were identified in this study. The investigation might provide useful information for future studies on genetics and immunity of *E. sinensis* and other economic crustaceans.

## Materials and Methods

### Ethic statement

All animal treatments of the study were strictly carried out according to the Guide for Care and Use of Laboratory Animals by Chinese Association for Laboratory Animal Sciences (No. 2011-2).

### Preparation of experimental crabs

Healthy berried female mitten crabs were obtained from a farm in Panjin, China and cultured in aerated seawater at 18±1 °C. During whole period of the experiment, all crabs were fed with clam meat once daily at night. Egg incubation and larval hatching were carried out using the same method with Sui et al [34]. In brief, berried crabs were incubated until larvae were hatched and newly hatched larvae were immediately challenged with microorganisms.

Three pathogen strains (Gram-negative bacteria *Vibrio alginolyticus*, Gram-positive bacteria *Micrococcus luteus* and fungi *Pichia pastoris*) were mixed and suspended in 0.1 mol/L PBS (pH 7.0) with the final pathogen concentration of 10^8^ cfu·mL^-1^. Lots of small newly hatched larvae were too hard to be directly injected into the body. It might also influence crab responses by physical stimulation as well. To overcome these shortcomings, hundreds of zoea larvae were cultured in seawater containing 100 μL the mixture of pathogens. At 1h post-challenge, whole bodies of all the treated larvae were collected with mesh grid and pooled as one sample for RNA isolation and transcriptome analysis. They were immediately placed in liquid nitrogen until use.

### cDNA preparation, transcriptome sequencing and assembly

Total RNA was extracted using Trizol Reagent (Invitrogen). RNA quality and concentration were determined by 1% agarose gel electrophoresis and a NanoDrop spectrophotometer. Polyadenylated mRNA was purified from total RNA using oligo(dT) magnetic beads and Oligotex mRNA Kits (Qiagen). They were fragmented by treating with heat and divalent cations before cDNA synthesis. The cDNA was reverse transcribed with random hexamer primers, end repaired by DNA polymerase and adapter ligated with T4 DNA ligase, according to Illumina manufacturer’s protocol.

Ligated products were PCR-amplified and sequenced from both 5' and 3' ends on an Illumina HiSeq 2000 platform. Raw data of Illumina sequencing were obtained after base calling and stored in fastq format. Cleaning steps of the raw reads were as follows: (1) trimming adapter sequences; (2) removing the reads that contain ambiguous ‘N’ nucleotides over 10%; (3) filtering the reads with more than 50% bases having a quality score lower than 5. All subsequent analyses were based on the remaining clean reads.

**Figure 1:**
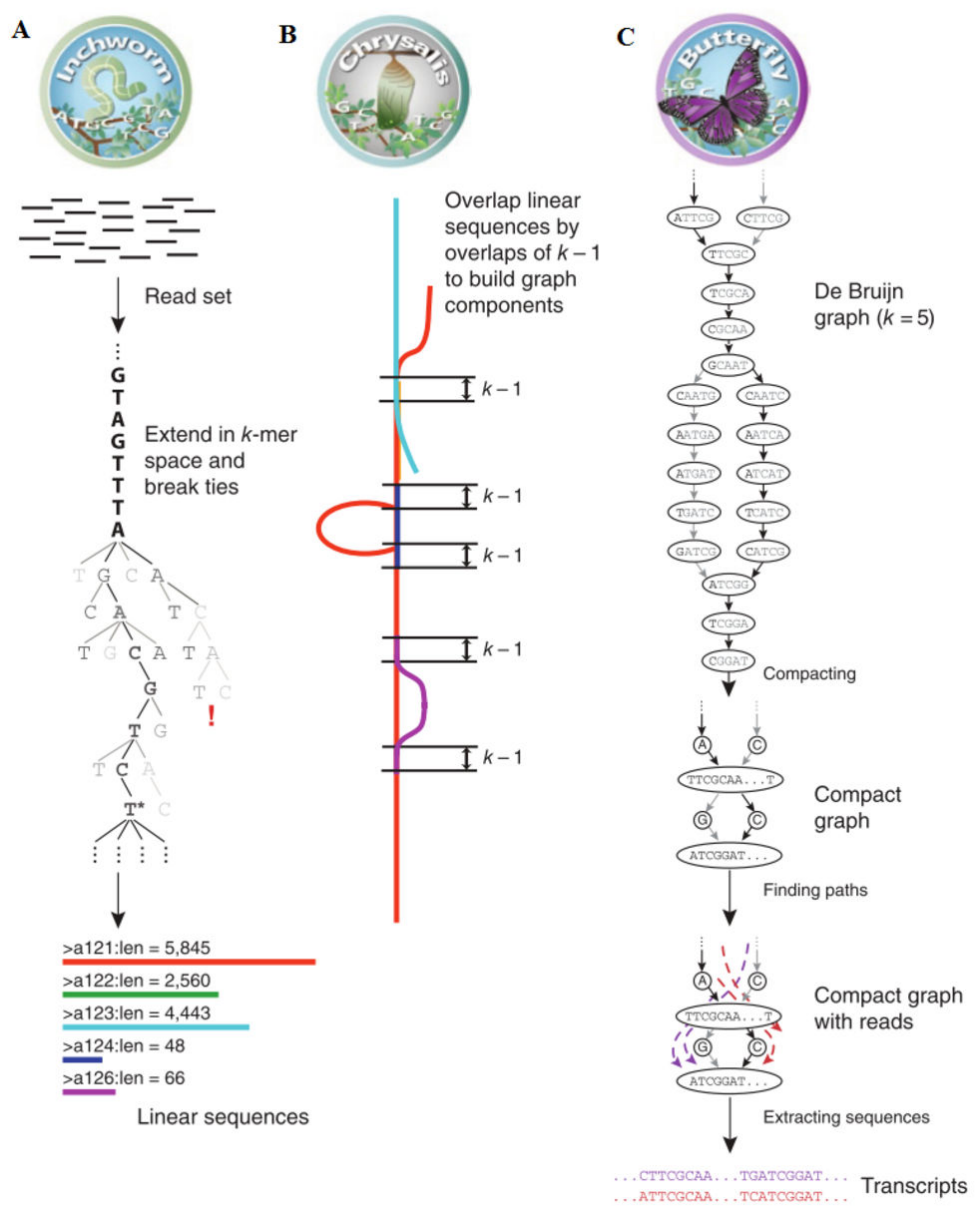
Overview of Trinity and workflow of *de* *novo* assembly. (a) Inchworm assembles RNA-seq data by searching for paths in a k-mer graph, generating linear contigs with each k-mer presents only once in the contigs. (b) Chrysalis clusters contigs if they share at least one *k*−1-mer and if reads span the junction between contigs, and then constructs individual de Bruijn graphs from each cluster. (c) Butterfly takes each de Bruijn graph from Chrysalis, followed by trimming spurious edges and compacting linear paths. It then traces the graph with reads and pairs, ultimately reporting linear sequence for each splice form and teasing paralogous transcript apart.

*De novo* assembly of full-length transcripts (Figure 1) was performed with Trinity software (http://trinityrnaseq.sf.net), referring to the strategy of Grabherr et al [35]. In general, Trinity was combined of three independent software modules: Inchworm, Chrysalis and Butterfly. It segmented sequence data to many individual de Bruijn graphs (each represented transcriptional complexity for a given gene) and processed every graph independently to extract full-length splicing isoforms and to output transcripts from paralogous genes (Figure 1). The k-mer value was set to 25 during this period. If a component had more than one transcript, the longest one was selected to represent assembled component in order to eliminate redundancy. To assess coverage of this transcriptome data, the assembled unigene dataset was compared with EST dataset that was available from NCBI Genbank (http://www.ncbi.nlm.nih.gov/nucest/?term=Eriocheir *sinensis*) using Blast program with an E-value threshold of 1E-5.

### Bioinformatics analysis and functional annotation

After being compared with NCBI EST dataset, transcriptomic dataset was analyzed using an established approach. Briefly, assembled unigenes were annotated using Blastx algorithm (*E*-value cut off: < 10^-10^) with public sequences in NCBI non-redundant protein (Nr), non-redundant nucleotide (Nt) databases (http://www.ncbi.nlm.nih.gov/), and UniProtKB/Swiss-Prot sequence database (http://www.ebi.ac.uk/uniprot/). Gene encoding protein domains were identified by searching against Protein Family (Pfam) database (http://pfam.janelia.org/) by means of hmmpfam program. Moreover, Gene Ontology (GO) (http://www.geneontology.org/) categorization was done with Blast2go program and WEGO software. Clusters of Orthologous Groups (COG) (http://www.ncbi.nlm.nih.gov/COG/) based analysis was then conducted to predict gene functions according to the known orthologous products. Using Enzyme Commission (EC) terms, biochemical pathway information was collected by downloading relevant maps from Kyoto Encyclopedia of Genes and Genomes (KEGG) (http://www.genome.jp/kegg/). Both COG and KEGG classifications were also performed using Blast algorithm.

### Gene discovery and SNP identification

Functional genes and molecular markers were deep investigated using the transcriptome data from all the zoea larvae. Presence and absence of immune relevant molecules were manually identified based on matched sequences in public databases [33]. For putative SNP detection, sequencing reads were mapped onto assembled unigenes with SOAPsnp software. Various parameters such as base quality score and read depth were optimized to identify final set of potential SNPs. Base quality score of ≥20 were set to assess the quality of reads at positions for SNP detection. Under the criteria of read depth of four and the minimum variant frequency of two, variations compared to the consensus sequence were counted as SNPs. Furthermore, they were considered statistically significant at a false discovery rate (FDR)/tested p-value <0.1.

## Results

### Transcriptome sequencing and assembly

**Figure 2:**
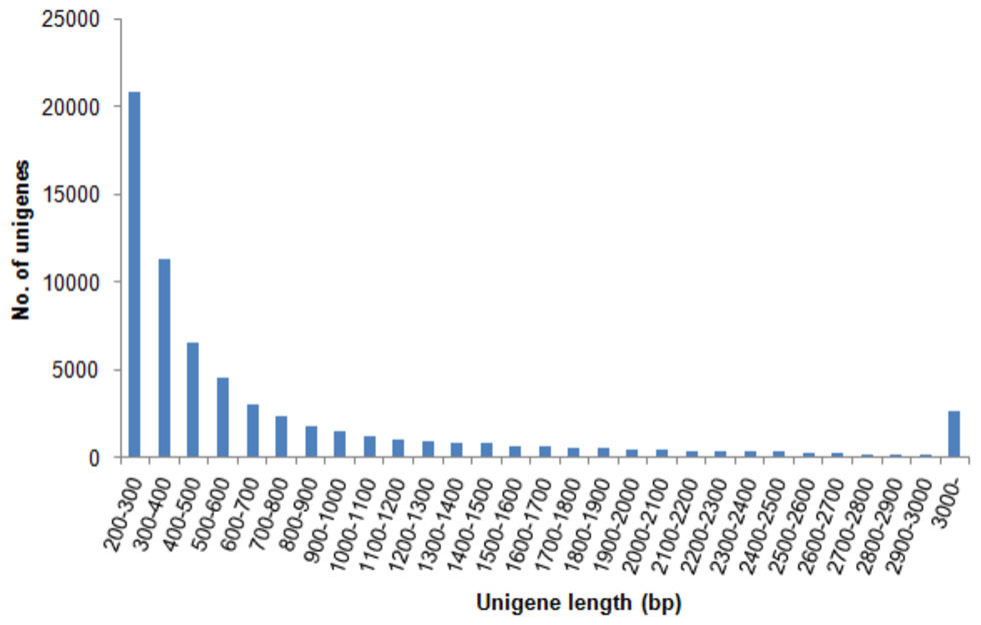
Length distribution of assembled unigenes for *E. sinensis* larvae transcriptome. The x-axis indicates unigene size and the y-axis indicates number of unigenes of each size.

Illumina sequencing-received raw data were deposited in NCBI short read archive database (accession number: SRA068379). Totally 46,099,408 raw reads were obtained from whole bodies of microbial challenged *E. sinensis* larvae (Table 1). After eliminating adapters, ambiguous nucleotides and low-quality sequences, 44,767,566 clean reads remained and they accumulated to be 4.52 Gb with a GC percentage of 47.00% (Table 1). Remaining clean reads were then assembled into 100,252 unigenes with a N50 length of 2,095 bp and an average size of 1,042 bp (Table 1). Assembled unigenes ranged from 201 bp to 19,357 bp and about half of them (51,156, 51.03%) were 200-500 bp in length (Figure 2). After elimination of repetition and short-length sequences, 65,535 non-redundant unigenes were selected for further analysis.

**Table 1:** Overview of *E. sinensis* larvae transcriptome sequencing and assembly.

Summary statistics	Number	
Total raw reads	46,099,408	
Total clean reads	44,767,566	
Total clean base pairs (Gb)	4.52	
Average length of clean reads (bp)	100.97	
GC percentage (%)	47.00	
Q20 percentage (%)	97.70	
Total number of unigenes	100,252	
Min**-**Max length of unigenes (bp)	201-19,357	
Average length of unigenes (bp)	1,042	
N50 of unigenes (bp)	2,095	
N90 of unigenes (bp)	372	

To evaluate coverage and abundance of this transcriptome data, assembled unigenes were compared against known EST sequences of *E. sinensis*. A total of 16,987 ESTs were downloaded from NCBI Genebank, of which 87.07% (14,790) were matched to transcriptome unigenes. However, only 9.49% (6,216 of 65,535) unigenes could be matched to NCBI ESTs (Table 2).

**Table 2:** Comparison between transcriptome non-redundant unigenes and NCBI ESTs of *E. sinensis*.

	Total number	Blast hit number	Coverage
ESTs	16,987	14,790	87.07%
Unigenes	65,535	6,216	9.49%

### Unigene annotation

**Figure 3:**
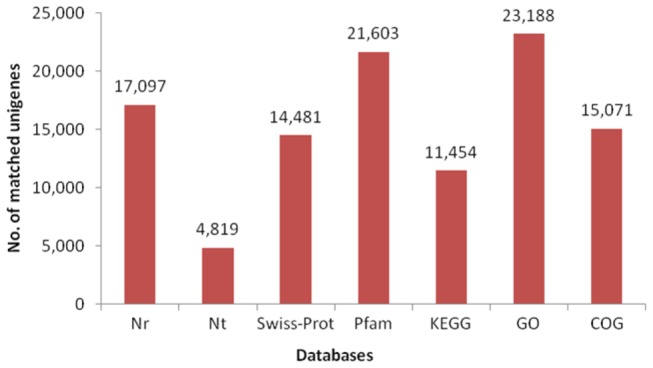
Summary statistics of Blast searches to annotated sequence databases.

**Figure 4:**
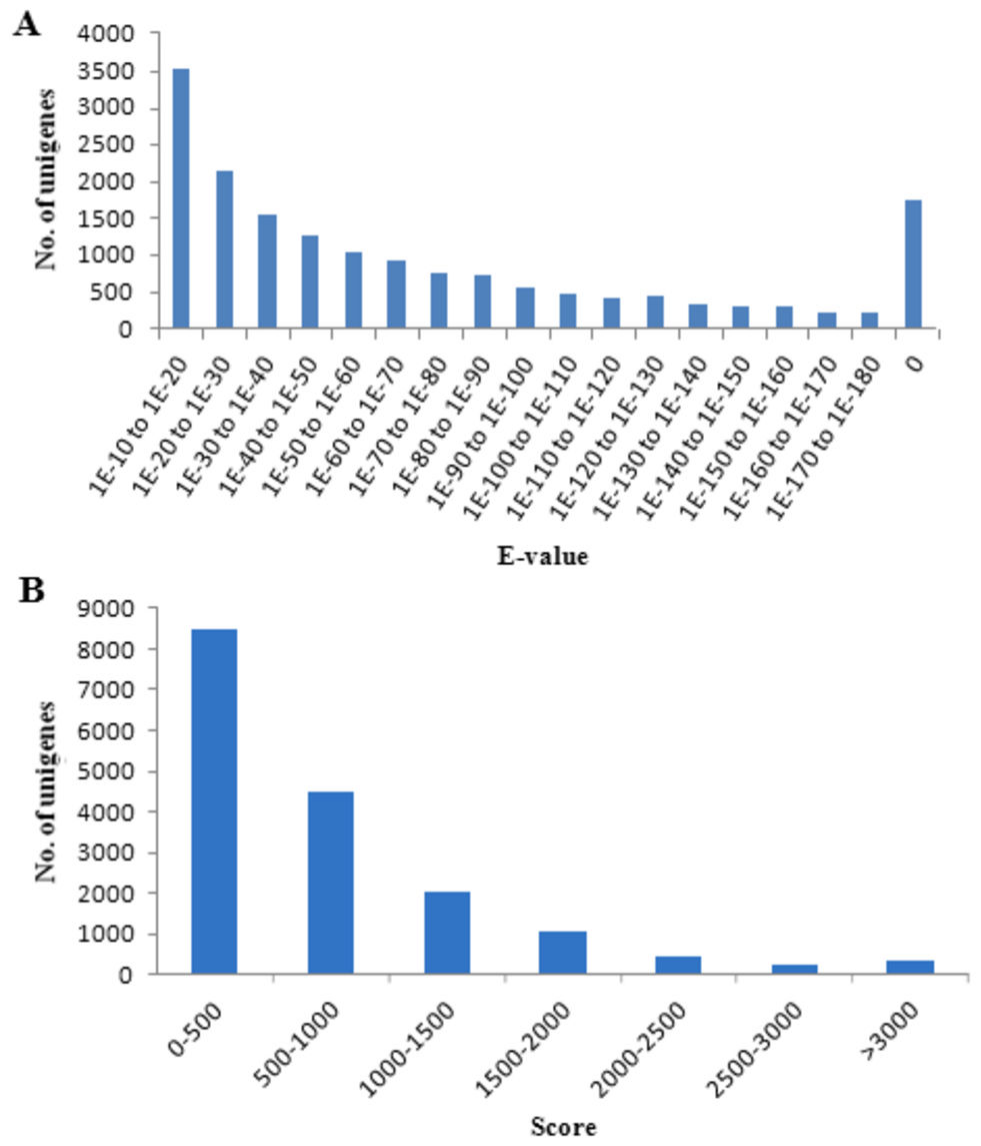
*E*-value and score distribution of *E. sinensis* larvae transcriptome unigenes with annotation to Nr database. (A) *E*-value distribution of annotated unigenes; (B) Score distribution of annotated unigenes.

To estimate putative functions of them, non-redundant unigenes were subjected to public databases for Blast analysis. Approximately 17,097 unigenes, which took up a proportion of 26.09%, showed significant blast hits against known sequences in Nr database (Figure 3). *E*-value distribution of matched sequences revealed that almost half of them (49.92%, 8,534) had an *E*-value from 1E-10 to 1E-50, while 10.24% (1,751) with the *E*-value to be zero (Figure 4A). Moreover, 26.28% (4,493) of them had a 500 to 1,000 score during the alignment with other sequences in Nr database, while 24.20% (4,138) had a larger score than 1,000 (Figure 4B). Apart from matched unigenes, the other 48,438 unigenes had no blast hits with any protein sequences in Nr database.

In addition, 4,819 (7.35%) unigenes were annotated in Nt database and 14,481 (22.10%) in Swiss-Prot database (Figure 3). Unigenes were then tested by querying against Pfam database for homologous domains and motifs. The query results indicated that 21,603 (32.96%) unigenes encoded similar protein domains to other sequences, while encoding domains of the other 43,932 (67.04%) unigenes were not found in any sequences or any species (Figure 3).

### Functional classification

Assembled non-redundant unigenes were also subjected to GO, COG and KEGG databases for blast searching. Summary statistics of them were shown in Figure 3.

**Figure 5:**
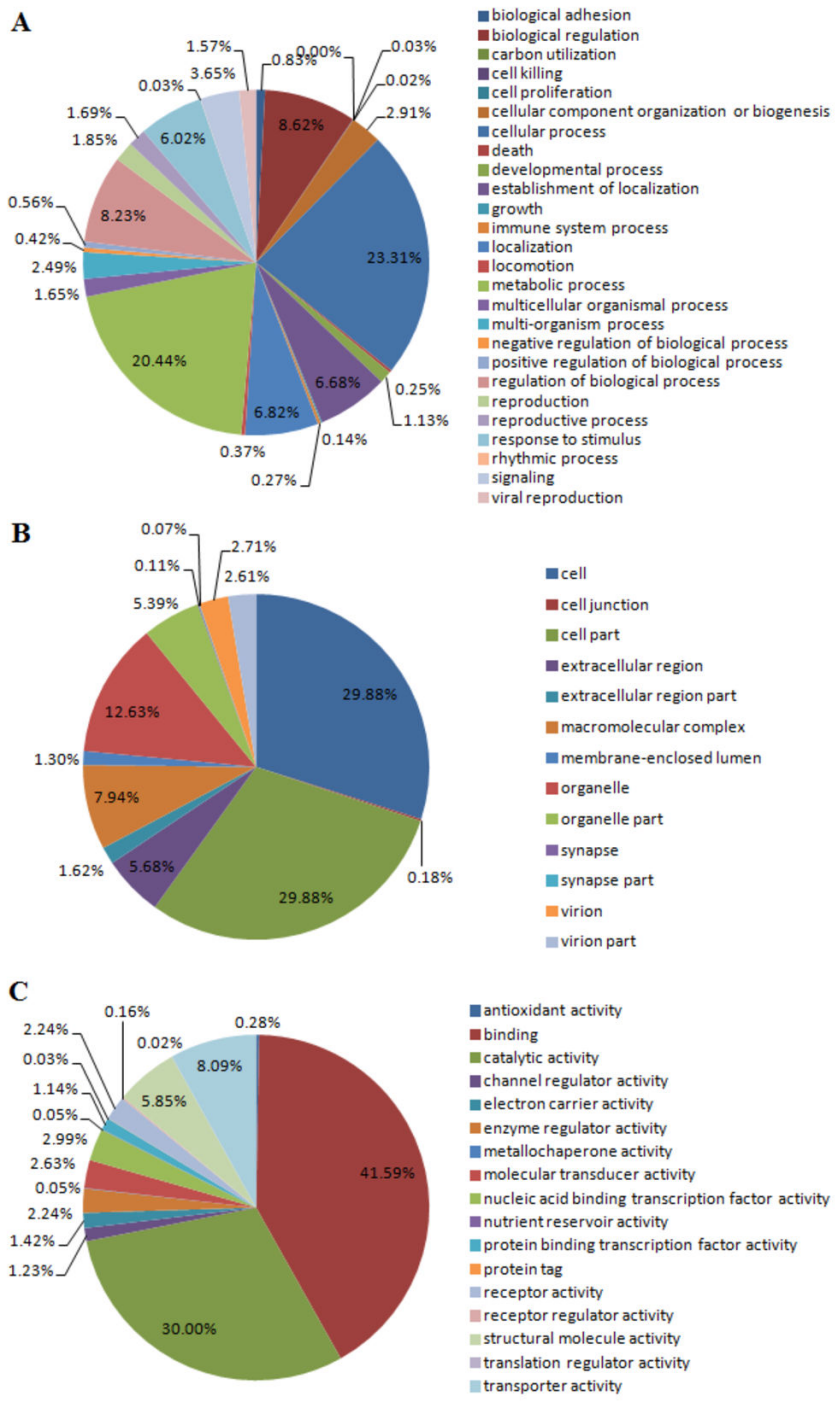
Distribution of Gene Ontology (GO) functional categories for *E. sinensis* larvae transcriptome. (A) Biological process; (B) Cellular component; (C) Molecular function. Each annotated sequence is assigned at least one GO term. All data are presented on the basis of GO second level terms. Numbers refer to percentage of assigned unigenes in each category.

GO is an international standardized gene functional classification system to comprehensively describe characteristics of different genes and their products. In this study, 23,188 unigenes were categorized by GO analysis (Figure 3). Second-level GO terms were applied to classify unigenes in terms of their involvement in three main categories (biological process, cellular component and molecular function) and each unigene was assigned at least one GO term. Twenty-six functional subcategories were grouped to biological process, among which ‘cellular process’ (23.31%) and ‘metabolic process’ (20.44%) contained the highest number of unigenes (Figure 5A). Seven subcategories were assigned into cellular component, of which ‘cell’ (29.88%) and ‘cell part’ (29.88%) were most dominant (Figure 5B). Seventeen subcategories were classified into molecular function category, among which the largest subcategory was ‘binding’ (41.59%) and ‘catalytic activity’ (30.00%) (Figure 5C).

**Figure 6:**
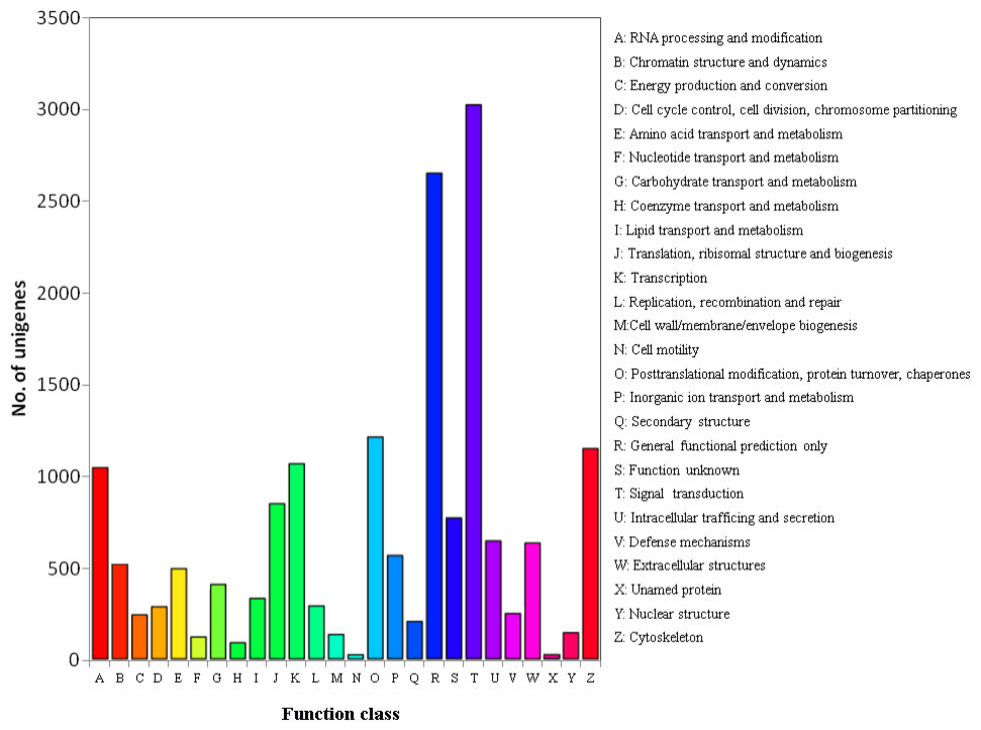
COG classification of putative proteins for *E. sinensis* larvae transcriptome.

COG database is a database in which orthologous gene products are classified. To further evaluate the completeness of our transcriptome library and the effectiveness of the annotation process, annotation of COG were selected and 15,071 unigenes were clustered in different processes (Figure 3). Five largest of the 26 COG categories were ‘signal transduction mechanisms’ (3,035), ‘general function prediction only’ (2,661), ‘post-translational modification, protein turnover, chaperon’ (1,221), ‘cytoskeleton’ (1,157) and ‘transcription’ (1083), while the three smallest clusters were ‘coenzyme metabolism’ (98), ‘cell’ (25) and ‘unnamed protein’ (22) (Figure 6).

KEGG pathway-based analysis facilitated systematical study on complicated metabolic pathways and biological behaviors of functional molecules. Thousands of unigenes were consequently classified into specific pathways (Table 3), among which most fell into ‘human diseases’ (2,880) and ‘metabolism’ (2,451), followed by ‘organism system’ (2,084), ‘genetic information processing’ (1,762) and ‘cellular processes’ (1,244), while least were assigned to ‘environmental information processing’ (1,033). Predominant subcategories of all the pathways were ‘infectious diseases’ (1205), ‘signal transduction’ (807) and ‘translation’ (753).

**Table 3:** KEGG assignment of non-redundant unigenes for *E. sinensis* larvae transcriptome.

KEGG category	KEGG subcategory	No. of unigenes
Metabolism	Amino acid metabolism	346
	Biosynthesis of other secondary metabolites	38
	Carbohydrate metabolism	455
	Energy metabolism	229
	Glycan biosynthesis and metabolism	307
	Lipid metabolism	352
	Metabolism of cofactors and vitamins	151
	Metabolism of other amino acids	125
	Metabolism of terpenoids and polyketides	43
	Nucleotide metabolism	282
	Xenobiotics biodegradation and metabolism	123
Genetic information processing	Folding	496
	Replication and repair	284
	Transcription	229
	Translation	753
Environmental information processing	Membrane transport	39
	Signal transduction	807
	Signaling molecules and interaction	187
Cellular processes	Cell communication	264
	Cell growth and death	379
	Cell motility	99
	Transport and catabolism	502
Organismal systems	Circulatory System	91
	Development	105
	Digestive System	334
	Endocrine System	354
	Environmental Adaptation	36
	Excretory System	128
	Immune System	408
	Nervous System	548
	Sensory System	80
Human diseases	Cancers	689
	Cardiovascular diseases	133
	Endocrine and metabolic diseases	20
	Immune diseases	72
	Infectious diseases	1,205
	Neurodegenerative diseases	484
	Substance dependence	277

### Annotation of immune-relevant genes and pathways

Using the transcriptome data as references, immune relevant genes, metabolic and signaling pathways were analyzed to gain deep insight into immune system of the crab. As shown in Figure 6, 3,292 unigenes were classified into COG categories of ‘signal transduction mechanisms’ and ‘defense mechanisms’. About 1,402 unigenes were highly enriched in KEGG subcategories of ‘immune system’, ‘signal transduction’ and ‘signaling molecules and interaction’ (Table 3). These results indicated considerable immune and transduction-related genes that were associated with various known metabolic or pathways. Lots of functional molecules involved in multiple immune pathways were then analyzed.

**Figure 7:**
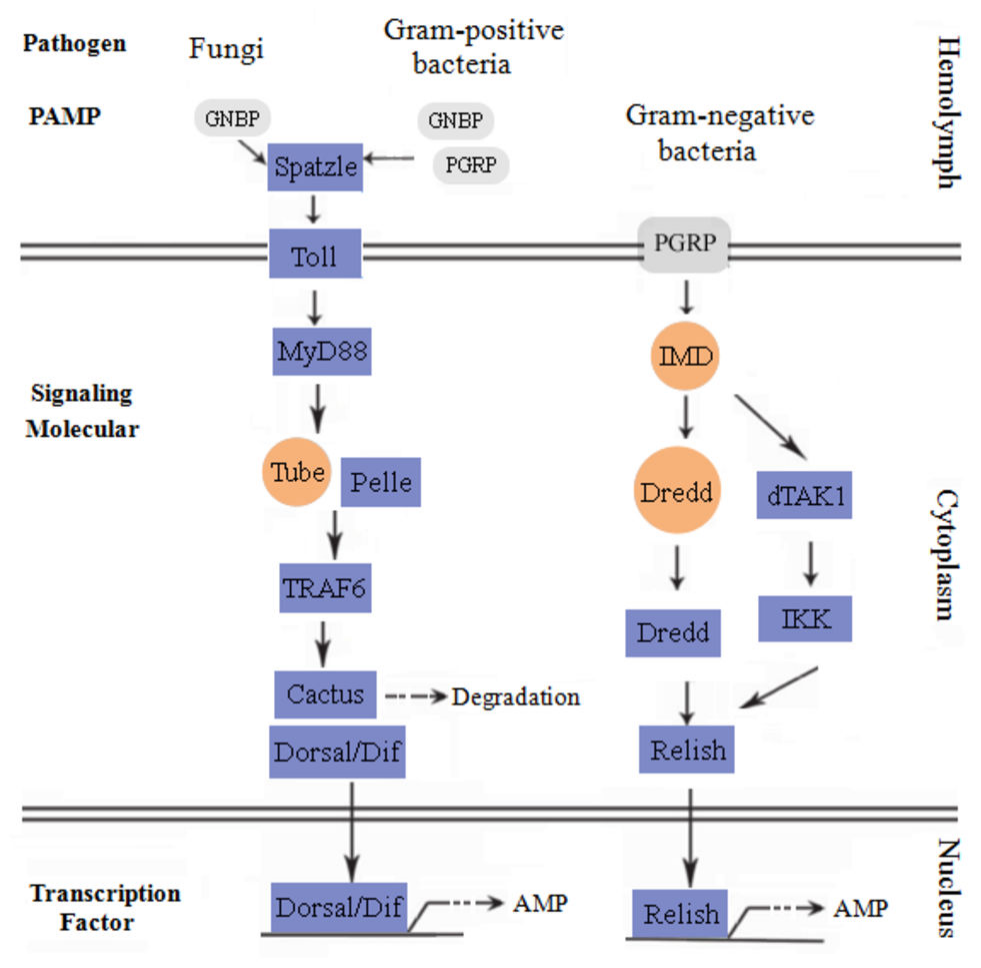
Putative Toll pathway and IMD pathway. Putative Toll and IMD pathways of *E. sinensis* were constructed based on the knowledge in *Drosophila*, shrimps and other species. Purple square indicated proteins that were identified in microbial challenged *E. sinensis* larvae; and orange circle, not identified. Most interactions have to be confirmed experimentally.

Well-studied signaling pathways involved in innate immunity are Toll pathway and IMD pathway, which actively participate in anti-bacterial processes. In the study, we found many key components of the two pathways, referring to the knowledge in *Drosophila melanogaster*, shrimps and other relative species [36-38]. Members of Toll pathway were mainly composed of Toll receptor, Spatzle and the corresponding adaptors such as myeloid differentiation factor 88 (Myd88), Pelle, tumor necrosis factor receptor associated factor 6 (TRAF6), Cactus and Dorsal/ Dorsal-related immunity factor (Dif) (Figure 7, Table S1). Key adaptor proteins of IMD pathway included transforming growth factor beta–activated kinase dTAK1, inhibitor of nuclear factor kappa-B kinase (IKK), Dredd/Caspase and the related nuclear transcription factor Relish (Figure 7, Table S2). Via Toll and IMD pathways, these molecules may induce the expression of their downstream effectors, antimicrobial peptide (AMP) genes [36].

**Figure 8:**
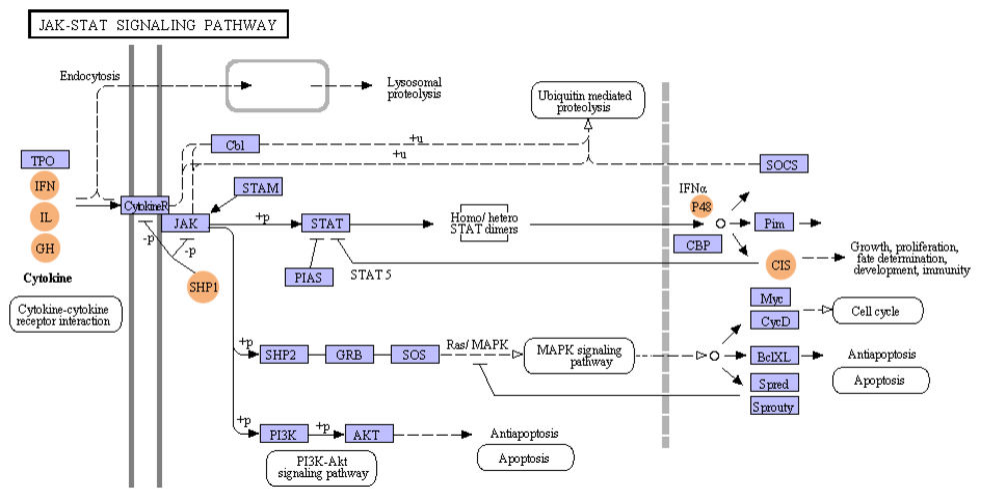
Putative JAK-STAT pathway. Putative JAK-STAT pathway of *E. sinensis* was constructed based on KEGG mapping. Purple square indicated proteins that were identified in microbial challenged *E. sinensis* larvae; and orange circle, not identified. Most interactions have to be confirmed experimentally.

**Figure 9:**
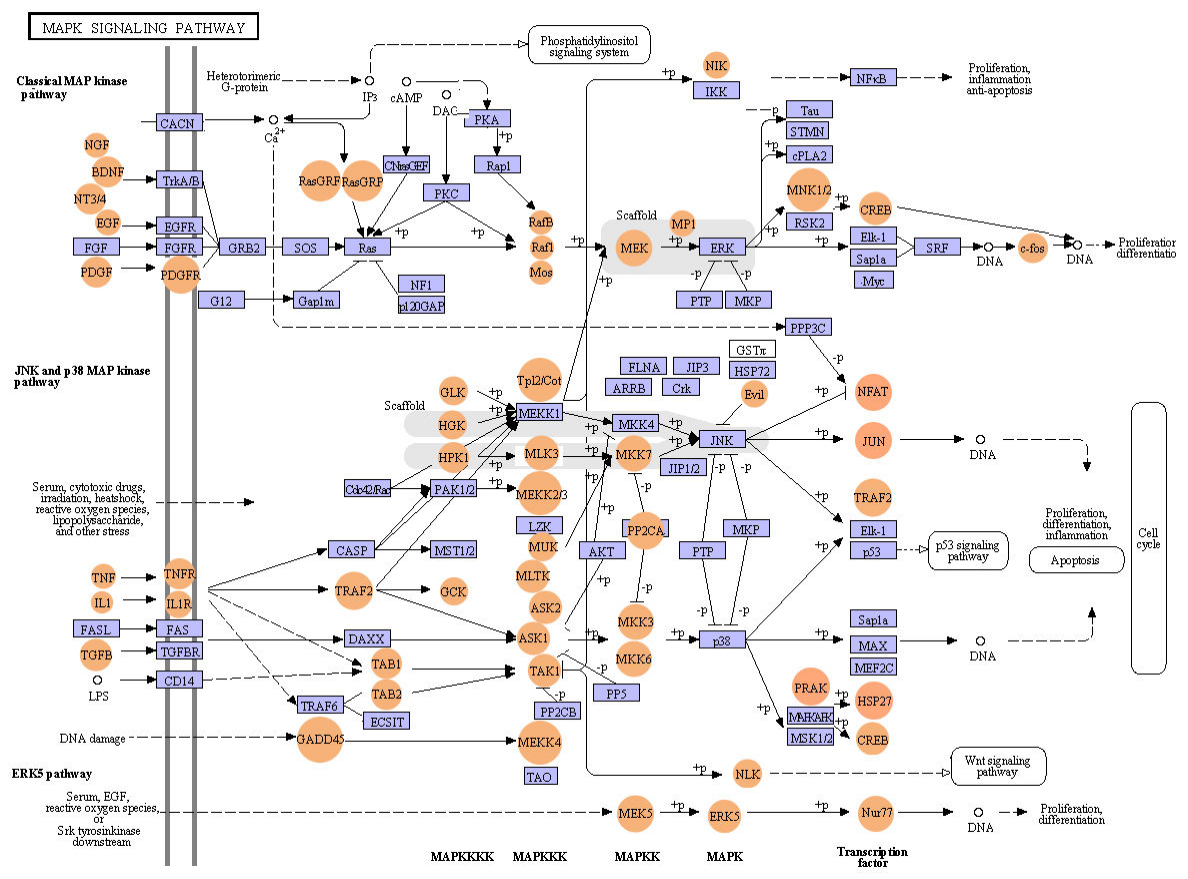
Putative MAPK pathway. Putative MAPK pathway of *E. sinensis* was constructed based on KEGG mapping. Purple square indicated proteins that were identified in microbial challenged *E. sinensis* larvae; and orange circle, not identified. Most interactions have to be confirmed experimentally.

Different members of Jak-Stat pathway and MAPK pathway were detected based on reference information of KEGG mapping. Major effectors involved in Jak-Stat pathway were cytokines, cytokine-receptors (CytokineR), JAK and STAT (Figure 8, Table S3). Their downstream regulatory molecules such as cytokine inducible SH2-containing protein (CIS), suppressor of cytokine signaling (SOCS), SH2-containing phosphatase, tyrosine-protein phosphatase non-receptor type 6 (SHP1), protein inhibitor of activated STAT (PIAS) and signal transducing adaptor molecule (STAM) were also detected (Table S3). In MAPK pathway, protein kinases could be grouped into three main families, including extracellular signal-regulated kinase (ERK), c-Jun N-terminal kinase (JNK) and p38/stress-activated protein kinase (p38/SAPK) (Figure 9, Table S4). We also found many other key members of the conserved protease cascades like MAPK kinase kinase kinase, MAPK kinase kinase/MEKK, MAPK kinase/MKK, and the activated transcription factors like p53, nuclear factor kappa-B (NF-κB), MAX protein and cyclic AMP-dependent transcription factor (ATF2) (Table S4). They may also play pivotal roles in many biological responses of mitten crab through putative Jak-Stat and MAPK pathways.

### SNP markers

**Figure 10:**
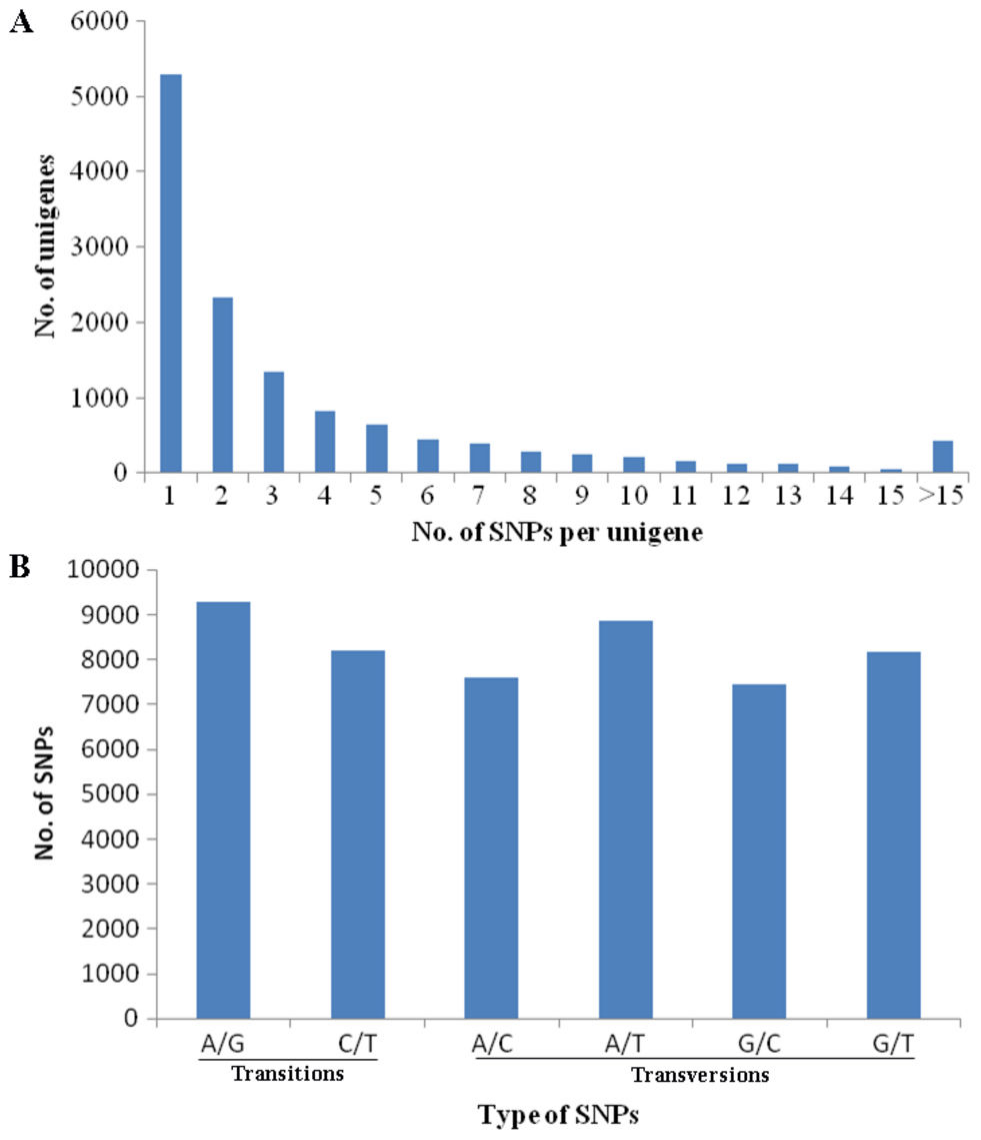
Distribution and classification of identified SNPs. (A) Number of SNPs distributed per unigene; (B) Classification of different substitution types of SNPs.

Putative SNPs were screened following specific criteria according to base quality score, read depth and minor allele frequency (see Materials and Methods). With these criteria, 49,555 putative SNPs were identified from 13,039 assembled unigenes (Table 4), which were identified with the FDR/p-value of 0.1. Average frequency of the SNPs was one SNP for every 244 bp (or 0.41 SNP per 100 bp). The number of SNPs per unigene was highly variable from one to fifty-three. Among all unigenes with identified SNPs, up to 40.56% contained only one SNP (Figure 10A). About 56.12% unigenes were detected with two to fifteen SNPs per unigene, while only a few (3.31%) had more than 15 (Figure 10A). 32,085 of all the putative SNPs were transversions (Tv) and 17,470 were transitions (Ts), with a mean ratio (Tv:Ts) of 1.84:1.00 across the transcriptome (Figure 10B). A/G substitutions were frequent and accounted for 18.73% of all SNPs (Figure 10B).

**Table 4:** Summary description of putative SNPs identified from *E. sinensis* larvae transcriptome.

Description	Number
Unigenes with SNPs	13,309
Total SNPs	49,555
Transitions	14,470
Transversions	32,085
bp/SNP	244
Average depth in SNP position (min-max)	614.60 (11-22276)

To analyze sequence variants of immune genes, 176 candidate SNPs from 38 unigenes were found to be involved with the four mentioned immune pathways (Table 5). The number of SNPs in every unigene ranged from one to 46 and most unigenes had only one SNP. Among all the 38 unigenes, Spatzle was found to contain largest number of SNPs, followed by cell division control protein 42/Ras-related C3 botulinum toxin substrate 1(cdc42/Rac), growth factor receptor-binding protein 2 (GRB2) and tumor protein P53 (Table 5).

**Table 5:** List of 176 SNPs in 38 immune pathway-related unigenes.

Gene category	Unigene component	No. of SNPs
Spatzle	comp33625_c0	14
	comp37644_c0	46
Toll	comp44024_c0	1
	comp45455_c0	3
Pelle	comp35625_c0	3
Relish	comp43492_c2	4
	comp43492_c3	6
Jak	comp42519_c2	4
Cyclin D	comp42339_c0	2
PI3K	comp419368_c0	1
Akt	comp43162_c2	1
CBP	comp45257_c2	1
SOCS	comp29409_c0	1
Spred	comp42402_c2	6
CACN	comp40211_c0	1
	comp42200_c0	5
EGFR	comp45184_c1	1
FGFR	comp39916_c0	1
GRB2	comp41990_c6	16
Ras	comp43121_c0	1
p120GAP	comp41773_c0	1
NF1	comp45632_c0	7
	comp45632_c1	2
ERK	comp36917_c0	4
PTP	comp27866_c0	1
MKP	comp36734_c1	1
STMN1	comp42386_c1	1
RSK2	comp43663_c1	1
PPP3C	comp33500_c0	1
FASL	comp44048_c0	1
DAXX	comp37820_c0	1
ECSIT	comp42144_c1	3
cdc42/Rac	comp103709_c0	17
	comp39531_c0	1
MKK4	comp38478_c0	1
P53	comp44289_c7	10
	comp45072_c0	2
ATF2	comp44533_c0	3

## Discussion

Knowledge of genetic information is essential for aquaculture management and sustainable development of crustacean fisheries. However, only genome of *Daphnia pulex* is sequenced in Crustacea [39]. Lack of fully sequenced genome not only limits genetic resources of crustacean, but also hampers researches on gene expression and the regulations. Fortunately, with development of EST method and high-throughput sequencing technology, some genes have been revealed from transcriptome analyses of the important crustaceans like *E. sinensis*. In this study, whole bodies of *E. sinensis* larvae after microbial challenge are used for the first time to analyze *E. sinensis* transcriptome and discover immune functional genes.

Previous transcriptome studies of *E. sinensis* have been performed from single organ and tissue of the crab [3,5,33,40-43]. Differently, our study covers all tissues of *E. sinensis* larvae and contains fuller transcriptional genes of the organism. It largely enriches transcriptional sources of mitten crab. Transcriptome data is known to be the completed RNA transcripts in a cell. Characterization of transcriptome is important to explain functional complexity of genome and to understand cell activities like growth, development, disease and immune response [44]. Therefore, our report offers a general view on gene background and immune system of the crab.

In detail, many ESTs are obtained from tissues like testis, haemocyte and hepatopancreas of *E. sinensis* by Sanger sequencing approach [3,5,40] and they all have been submitted to NCBI. Our analysis shows that only 12.93% NCBI ESTs could not be matched to transcriptome unigenes of *E. sinensis* larvae, while up to 90.51% unigenes could not matched to NCBI ESTs of *E. sinensis*. It implies deep coverage of *E. sinensis* larval transcriptome and supplies considerable gene resources of the crab. In comparison, 60.1% NCBI ESTs from muscle, blood, hepatopancreas and other organs of *L. vannamei* are matched to transcriptome unigenes in *L. vannamei* larvae, whereas 85.8% of larvae unigenes are not matched to NCBI ESTs [28]. Our results are similar with the report of *L. vannamei* and greatly enrich transcriptional data of important economic crustaceans. Additionally, Pfam searching of protein homologous domain/motif shows many genes without blast hits in any species. It will be helpful to explore new sequences and study on them, such as molecular characterization, sequence structure analysis, expression pattern analysis and biological activity test.

The research shows that infection with various microbes, including Gram-positive bacteria *Micrococcus luteus*, Gram-negative bacteria *Vibrio alginolyticus* and fungi *Pichia pastoris*, can help to acquire abundant information of immune genes. A considerable amount of genes relating to Toll, IMD, JAK-STAT and MAPK pathways are fully and systematically characterized in our study. Most genes are also detected in infected *Fenneropenaeus chinensis* pleopod and *E. sinensis* hepatopancreas [29,33]. Those pathways and genes play important roles in signal transduction, immune defense and other responses [33]. Hence, the study serves a good idea to identify functional genes and understand host immunity mechanism in crustacean at whole transcriptome level.

Comparing with previous transcriptome data of *E. sinensis* [33], several molecules like TRAF6, fibroblast growth factor (FGF), protein tyrosine phosphatase (PTP) and JNK-interacting protein 1 (JIP1) are first found of existence in this study. TRAF6 is even detected in crab for the first time. It is the only molecule of TRAF family that functions as signal transducer for tumor necrosis factor receptor (TNFR) and interleukin-1 receptor (IL-1R)/Toll-like receptor (TLR) families [45]. It is also reported to be important in antibacterial and antiviral responses through immune pathways [45,46]. As expected, we find that expression of TRAF6 is involved in both Toll and MAPK signaling pathways of *E. sinensis* larvae. Moreover, the molecule is recently characterized to be a new STAT3 interactor and negatively regulate activation of JAK-STAT signaling pathway [47]. All the findings imply that TRAF6 has crucial and complicated role in immune system. In addition, several molecules like Tube, IMD and Dredd were not found in this transcriptome analysis. Fortunately, researches reveal that deep sequencing (such as *de novo* sequencing and resequencing) of the genome offers another strategy to find candidate functional genes [48,49]. With development of genome sequencing in crab, we may make further efforts to identify those genes.

In this analysis, although high data output is produced, only a few unigenes have blast hits in public databases. Sequences that are not definitively annotated possibly represent genes of unknown functions in *E. sinensis*. Alternatively, it may be because of the complicated gene background of *Eriocheir* species or other crustaceans and their limited sequence information. Besides, there might be some differences on gene sequences of the crab and other animal species. Considering these, it is quite common that many unigenes cannot be matched. With more crab genetic information being studied and high-throughput sequencing data being applied, sequences obtained in the study will be further annotated and characterized.

Potential genes and pathways are also annotated in other larval transcriptome researches of arthropod species, including *G. mellonella* [32], *Bactrocera dorsalis* [50] and *Spodoptera exigua* [51]. The pathways are quite similar in different arthropods, which finally activate expression of AMPs and other proteins through interacting with NF-κB factors. Combing these similar reports in arthropod, annotated information is worthy of in-depth characterizing, which will facilitate researches on genetics, gene expression and regulation of whole bodies of their larvae.

Another important application of high-throughput sequencing technology is to identify genetic variants. It has been well established and demonstrated using Illumina technique to detect SNP mutations [52-54]. Our large-scale sequencing effort reveals lots of SNPs in *E. sinensis* larval transcriptome sequences. In the analysis, SNP types of A/G and C/T are quite common and SNP densities vary among different genes. It may be partly due to the relative functional importance of individual genes and the effects of selection [55]. Discovery of 49,555 SNPs, especially the 13,039 SNPs from immune pathway related genes, will therefore provide a valuable resource of candidate markers for future selective breeding of *E. sinensis*.

Many SNPs are also derived in other crustacean species with EST sequences through traditional sequencing method [54,56]. Both of the EST-SNP and high-throughput sequencing-derived SNP may lead to different alterations in amino acids and promote marker development of cultured crustaceans. However, total number of SNPs yielded in this study are much more than that of EST-derived SNPs in other crustacean studies [54,56]. It suggests high efficiency of high-throughput transcriptome analysis to gain SNP markers. In addition, our study is consistent with the report of catfish transcriptome that most unigenes had only one SNP [52]. It shows that most common genes have the same SNP density. However, Spatzle gene is found with largest number of SNPs in this study. Previous researches implies that Spatzle plays crucial role in recognizing pathogen associated molecular patterns and activating Toll receptor to initiate Toll pathway [57,58]. Application of these SNPs in Spatzle may be of great value in regulating signal transduction and antibacterial response of the crab.

## Conclusion

High-throughput sequencing technology offers a powerful approach to analyze gene expression and SNP markers in genome reference-free organisms. Using Illumina platform and *de novo* assembly technique, we have derived a dataset from whole bodies of *E. sinensis* larvae after microbial challenge. This data comprised 44,767,566 clean reads and 100,252 assembled unigenes. Enormous functional genes are detected to be related with multiple immune pathways, including Toll, IMD, JAK-STAT and MAPK pathways. Some important genes, including TRAF6, FGF, PTP and JIP1, are identified in *E. sinensis* for the first time. Particularly, TRAF6 is even first found of existence in crabs. 49,555 putative SNPs are also identified from the transcriptome data, which are useful to marker assisted selection of new strains in *E. sinensis*. Collectively, this is the first transcriptome report of microbial challenged *E. sinensis* larvae and it will provide valuable data to research immune mechanism and molecular biological of the crab.

## Supporting Information

Table S1 **Putative immune genes involved in Toll pathway of *E. sinensis* larvae.**
(DOC)

Table S2 **Putative immune genes involved in IMD pathway of *E. sinensis* larvae.**
(DOC)

Table S3 **Putative immune genes involved in JAK-STAT pathway of *E. sinensis* larvae.**
(DOC)

Table S4 **Putative immune genes involved in MAPK pathway of *E. sinensis* larvae.**
(DOC)
