# Supplementary material for: Transcriptome Profiling Analysis on Whole Bodies of Microbial Challenged Eriocheir sinensis Larvae for Immune Gene Identification and SNP Development
Source: PLoS One. 2013 Dec 4;8(12):e82156. doi: 10.1371/journal.pone.0082156 (PMC3852986; doi:10.1371/journal.pone.0082156)
Supplement: Table S1 — Putative immune genes involved in Toll pathway of E. sinensis larvae. (DOC) [file pone.0082156.s001.doc]

Table S1

Putative immune genes involved in Toll pathway of *E. sinensis* larvae

| Signaling molecular | Unigenes | ID | E-value | Description |
| --- | --- | --- | --- | --- |
| Spatzle | comp29177_c0 | gi|342326199|gb|AEL23015.1| | 2.97E-32 | protein spaetzle [*Cherax quadricarinatus*] |
|  | comp33625_c0 | gi|342326199|gb|AEL23015.1| | 2.97E-32 | protein spaetzle [*Cherax quadricarinatus*] |
|  | comp24164_c0 | gi|307191644|gb|EFN75124.1| | 8.67E-47 | Protein spaetzle [*Harpegnathos saltator*] |
|  | comp278682_c0 | gi|187764371|gb|ACD36030.1| | 5.40E-37 | spatzle protein [*Fenneropenaeus chinensis*] |
|  | comp37644_c0 | gi|187764371|gb|ACD36030.1| | 1.15E-27 | spatzle protein [*Fenneropenaeus chinensis*] |
| Toll | comp140609_c0 | gi|157107508|ref|XP_001649813.1| | 9.09E-13 | toll [*Aedes aegypti*] |
|  | comp11100_c0 | gi|165932241|dbj|BAF99007.1| | 1.33E-42 | toll receptor [*Marsupenaeus japonicus*] |
|  | comp11100_c1 | gi|165932241|dbj|BAF99007.1| | 1.33E-42 | toll receptor [*Marsupenaeus japonicus*] |
|  | comp44715_c0 | gi|165932241|dbj|BAF99007.1| | 0 | toll receptor [*Marsupenaeus japonicus*] |
|  | comp31681_c0 | gi|118421165|gb|ABK88278.1| | 3.33E-17 | toll-like receptor [*Carcinoscorpius rotundicauda*] |
|  | comp44024_c0 | gi|91076464|ref|XP_971999.1| | 0 | PREDICTED: similar to toll [*Tribolium castaneum*] |
|  | comp45455_c0 | gi|326927113|ref|XP_003209739.1| | 5.18E-43 | PREDICTED: toll-like receptor 13-like [*Meleagris gallopavo*] |
| MyD88 | comp37925_c0 | gi|307170866|gb|EFN62977.1| | 1.53E-52 | Myeloid differentiation primary response protein MyD88 [*Camponotus floridanus*] |
| Pelle | comp35625_c0 | gi|321474115|gb|EFX85081.1| | 3.21E-89 | *Litopenaeus vannamei* pelle mRNA, complete cds |
| TRAF6 | comp13680_c0 | ADM26237.1 | 2.45E-21 | tumor necrosis factor receptor-associated factor 6 [*Litopenaeus vannamei*] |
|  | comp16200_c0 | EFX81716.1 | 4.21E-139 | hypothetical protein DAPPUDRAFT_49933 [*Daphnia pulex]* |
|  | comp34241_c1 | EFX81716.1 | 4.21E-139 | hypothetical protein DAPPUDRAFT_49933 [*Daphnia pulex]* |
|  | comp40743_c0 | XP_002426151.1 | 3.07E-60 | TNF receptor-associated factor, putative [*Pediculus humanus corporis*] |
| Cactus | comp150341_c0 | gi|341650463|gb|JN180645.1| | 4.34E-13 | PREDICTED: tonsoku-like protein-like isoform 1 [*Sus scrofa*] |
|  | comp162911_c0 | gi|311253262|ref|XP_001927565.2| | 3.02E-47 | PREDICTED: tonsoku-like protein-like isoform 1 [*Sus scrofa*] |
| Dorsal/Dif | comp33537_c0 | gi|273548606|gb|ACZ98167.1| | 0 | dorsal [*Litopenaeus vannamei*] |
|  | comp22066_c0 | ADO17754.1 | 2.76E-17 | short gastrulation protein [*Parhyale hawaiensis*] |
|  | comp37252_c0 | ADO17754.1 | 7.09E-178 | short gastrulation protein [*Parhyale hawaiensis*] |
