# Supplementary material for: Transcriptome Profiling Analysis on Whole Bodies of Microbial Challenged Eriocheir sinensis Larvae for Immune Gene Identification and SNP Development
Source: PLoS One. 2013 Dec 4;8(12):e82156. doi: 10.1371/journal.pone.0082156 (PMC3852986; doi:10.1371/journal.pone.0082156)
Supplement: Table S2 — Putative immune genes involved in IMD pathway of E. sinensis larvae. (DOC) [file pone.0082156.s002.doc]

Table S2

Putative immune genes involved in IMD pathway of *E. sinensis* larvae

| Signaling molecular | Unigenes | ID | E-value | Description |
| --- | --- | --- | --- | --- |
| dTAK1 | comp42183_c1 | gi|241618065|ref|XP_002408296.1| | 1.02E-21 | mitogen activated protein kinase kinase kinase 1, MAPKKK1, MEKK1, putative [*Ixodes scapularis*] |
| IKK | comp37279_c0 | gi|2961203|gb|AAC05683.1| | 5.60E-56 | I-kappa-B kinase [*Crassostrea gigas*] |
| Dredd/ Casp | comp34703_c0 | XP_003385047.1 | 1.33E-20 | PREDICTED: caspase-3-like [*Amphimedon queenslandica*] |
|  | comp165136_c0 | ADH94015.1 | 8.26E-63 | caspase [Marsupenaeus japonicus] |
|  | comp287751_c0 | ADM45311.1 | 3.38E-27 | caspase [Eriocheir sinensis] |
| Relish | comp43492_c1 | gi|303307787|gb|ADM14334.1| | 4.60E-115 | relish [*Eriocheir sinensis*] |
|  | comp43492_c2 | gi|303307787|gb|ADM14334.1| | 0 | relish [*Eriocheir sinensis*] |
|  | comp43492_c3 | gi|303307787|gb|ADM14334.1| | 1.03E-168 | relish [*Eriocheir sinensis*] |
