# Supplementary material for: Transcriptome Profiling Analysis on Whole Bodies of Microbial Challenged Eriocheir sinensis Larvae for Immune Gene Identification and SNP Development
Source: PLoS One. 2013 Dec 4;8(12):e82156. doi: 10.1371/journal.pone.0082156 (PMC3852986; doi:10.1371/journal.pone.0082156)
Supplement: Table S3 — Putative immune genes involved in JAK-STAT pathway of E. sinensis larvae. (DOC) [file pone.0082156.s003.doc]

Table S3

Putative immune genes involved in JAK-STAT pathway of *E. sinensis* larvae

| Signaling molecular | Unigenes | ID | E-value | Description |
| --- | --- | --- | --- | --- |
| TPO | comp40184_c0 | XP_002431634.1 | 0 | Thyroid peroxidase precursor, putative [*Pediculus humanus corporis*] |
| CytokinR | comp43402_c1 | EFN76806.1 | 1.02E-75 | Cytokine receptor [*Harpegnathos saltator*] |
|  | comp12725_c0 | EGI63440.1 | 7.52E-16 | Cytokine receptor [*Acromyrmex echinatior*] |
|  | comp210974_c0 | EGI63440.1 | 7.52E-16 | Cytokine receptor [*Acromyrmex echinatior*] |
| JAK | comp30771_c0 | XP_002425471.1 | 3.44E-49 | tyrosine-protein kinase jak2, putative [*Pediculus humanus corporis*] |
|  | comp42519_c2 | XP_002425471.1 | 5.67E-122 | tyrosine-protein kinase jak2, putative [*Pediculus humanus corporis*] |
| STAT | comp35700_c0 | ACA79939.1 | 0 | STAT long form [*Penaeus monodon*] |
| STAM | comp42887_c5 | XP_003398833.1 | 1.29E-104 | PREDICTED: signal transducing adapter molecule 1-like [*Bombus terrestris*] |
| CBL | comp32967_c0 | XP_002428625.1 | 0 | E3 ubiquitin-protein ligase CBL, putative [*Pediculus humanus corporis*] |
| PIAS | comp387_c1 | NP_001072455.1 | 1.47E-26 | protein inhibitor of activated STAT, 2 [*Xenopus (Silurana) tropicalis*] |
|  | comp32289_c0 | EFN82639.1 | 5.21E-134 | E3 SUMO-protein ligase PIAS2 [*Harpegnathos saltator*] |
| CycD | comp16117_c0 | NP_001089817.1 | 1.95E-55 | cyclin D2 [*Xenopus laevis*] |
|  | comp42339_c0 | XP_974376.1 | 5.44E-50 | PREDICTED: similar to cyclin d [*Tribolium castaneum*] |
| SHP2 | comp28562_c0 | XP_002430772.1 | 2.00E-18 | tyrosine-protein phosphatase corkscrew, putative [*Pediculus humanus corporis*] |
|  | comp217328_c0 | XP_002430772.1 | 2.00E-18 | tyrosine-protein phosphatase corkscrew, putative [*Pediculus humanus corporis*] |
| GRB2 | comp15537_c0 | XP_969148.2 | 4.25E-66 | PREDICTED: similar to AGAP005258-PA [*Tribolium castaneum*] |
| SOS | comp41420_c4 | XP_002428152.1 | 0 | ras GTP exchange factor, son of sevenless, putative [*Pediculus humanus corporis*] |
| PI3K | comp45568_c0 | ADE44091.1 | 0 | phosphoinositide 3-kinase isoform b [*Panulirus argus*] |
|  | comp419368_c0 | ADE44090.1 | 2.46E-42 | phosphoinositide 3-kinase isoform a [*Panulirus argus*] |
|  | comp26230_c0 | XP_001606345.1 | 0 | PREDICTED: similar to MGC80357 protein [*Nasonia vitripennis*] |
| Akt | comp43162_c2 | ADM87425.3 | 0 | Akt [*Gecarcinus lateralis*] |
| CBP | comp33288_c0 | EFN64132.1 | 1.26E-88 | CREB-binding protein, putative [*Pediculus humanus corporis*] |
|  | comp45257_c2 | EFN64132.1 | 6.83E-111 | CREB-binding protein [*Camponotus floridanus*] |
| SOCS | comp1253_c0 | BAI70368.1 | 1.76E-11 | suppressor of cytokine signaling-2 like protein [*Marsupenaeus japonicus*] |
|  | comp29409_c0 | EGI69666.1 | 2.54E-85 | Suppressor of cytokine signaling 5 [*Acromyrmex echinatior*] |
|  | comp564240_c0 | BAI70368.1 | 1.79E-16 | suppressor of cytokine signaling-2 like protein [*Marsupenaeus japonicus*] |
|  | comp35667_c0 | ACU42699.1 | 0 | cytokine signaling 2 [*Eriocheir sinensis*] |
|  | comp42167_c0 | EEC10036.1 | 3.49E-62 | SOCS box SH2 domain-containing protein [*Ixodes scapularis*] |
|  | comp45440_c0 | XP_001603336.1 | 8.47E-94 | PREDICTED: similar to CG8146-PA [*Nasonia vitripennis*] |
| Pim | comp504845_c0 | XP_001827749.1 | 7.67E-77 | alpha-tubulin suppressor-like protein [*Enterocytozoon bieneusi H348*] |
|  | comp41596_c6 | NP_001090165.1 | 1.67E-89 | pim-3 oncogene [*Xenopus laevis*] |
| Myc | comp42818_c4 | EFX79343.1 | 9.02E-16 | Myc, dMyc-like protein [*Daphnia pulex*] |
|  | comp38595_c2 | EEZ99541.1 | 2.98E-15 | hypothetical protein TcasGA2_TC000123 [*Tribolium castaneum*] |
| BclXL | comp44609_c0 | EGI69168.1 | 2.17E-20 | Bcl-2-like protein 1 [*Acromyrmex echinatior*] |
| Sprouty | comp28930_c0 | EFZ18471.1 | 4.90E-45 | hypothetical protein SINV_11790 [*Solenopsis invicta*] |
| Spred | comp42402_c2 | NP_001164144.1 | 1.05E-46 | sprouty-related protein with EVH-1 domain [*Tribolium castaneum*] |
|  | comp42402_c3 | XP_002414876.1 | 3.24E-41 | sprouty protein evh1 domain-containing protein, putative [*Ixodes scapularis*] |
