# Supplementary material for: Transcriptome Profiling Analysis on Whole Bodies of Microbial Challenged Eriocheir sinensis Larvae for Immune Gene Identification and SNP Development
Source: PLoS One. 2013 Dec 4;8(12):e82156. doi: 10.1371/journal.pone.0082156 (PMC3852986; doi:10.1371/journal.pone.0082156)
Supplement: Table S4 — Putative immune genes involved in MAPK pathway of E. sinensis larvae. (DOC) [file pone.0082156.s004.doc]

Table S4

Putative immune genes involved in MAPK pathway of *E. sinensis* larvae

| Signaling molecular | Unigene | ID | E-value | Description |
| --- | --- | --- | --- | --- |
| CACN | comp17791_c0 | XP_002168351.1 | 3.42E-15 | PREDICTED: similar to calcium channel, voltage-dependent, alpha2/delta subunit 1, partial [Hydra magnipapillata] |
|  | comp182440_c0 | XP_001604402.1 | 1.78E-12 | PREDICTED: similar to voltage- gated calcium channel alpha subunit cav3.3 (voltage-dependent t-type calcium channel [Nasonia vitripennis] |
|  | comp233355_c0 | XP_002742061.1 | 1.68E-12 | PREDICTED: calcium channel, voltage-dependent, alpha2/delta subunit 3-like [Saccoglossus kowalevskii] |
|  | comp231730_c0 | XP_002408563.1 | 8.49E-19 | voltage-gated channel, putative [Ixodes scapularis] |
|  | comp3184_c0 | EFN60368.1 | 1.86E-41 | Voltage-dependent T-type calcium channel subunit alpha-1H [Camponotus floridanus] |
|  | comp33834_c0 | XP_001661548.1 | 2.24E-26 | voltage-dependent p/q type calcium channel [Aedes aegypti] |
|  | comp38909_c0 | EGI61013.1 | 7.94E-13 | Voltage-dependent T-type calcium channel subunit alpha-1G [Acromyrmex echinatior] |
|  | comp40211_c0 | XP_001807530.1 | 8.88E-45 | PREDICTED: similar to voltage-gated calcium channel alpha 1 subunit [Tribolium castaneum] |
|  | comp42200_c0 | XP_003251102.1 | 0 | PREDICTED: voltage-dependent calcium channel subunit alpha-2/delta-3-like [Apis mellifera] |
|  | comp42749_c0 | XP_003394404.1 | 4.91E-21 | PREDICTED: voltage-dependent calcium channel type A subunit alpha-1-like [Bombus terrestris] |
|  | comp43763_c0 | XP_001812120.1 | 0 | PREDICTED: similar to voltage-gated ion channel [Tribolium castaneum] |
|  | comp45001_c0 | ABL10360.2 | 0 | voltage-gated sodium channel [Cancer borealis] |
| FGF | comp168399_c0 | NP_001098748.1 | 4.81E-13 | fibroblast growth factor 1 (acidic) [Danio rerio] |
| EGFR | comp45184_c1 | XP_003395927.1 | 0 | PREDICTED: epidermal growth factor receptor-like [Bombus terrestris] |
| FGFR | comp20809_c0 | CAH03726.1 | 1.41E-49 | TPA: FGF receptor-like protein 1a [Takifugu rubripes] |
|  | comp31353_c0 | CAH03726.1 | 1.41E-49 | TPA: FGF receptor-like protein 1a [Takifugu rubripes] |
|  | comp32520_c0 | NP_001012263.2 | 1.30E-22 | fibroblast growth factor receptor-like 1b [Danio rerio] |
|  | comp39916_c0 | XP_003401483.1 | 0 | PREDICTED: fibroblast growth factor receptor homolog 1-like [Bombus terrestris] |
| GRB2 | comp41990_c6 | EGI65421.1 | 1.68E-128 | Insulin-like growth factor 2 mRNA-binding protein 1 [Acromyrmex echinatior] |
| SOS | comp41420_c4 | XP_002428152.1 | 0 | ras GTP exchange factor, son of sevenless, putative [Pediculus humanus corporis] |
| Ras | comp43121_c0 | AAK14389.1 | 1.34E-89 | Ras [Marsupenaeus japonicus] |
|  | comp5524_c0 | XP_001661022.1 | 7.26E-54 | MRAS2, putative [Aedes aegypti] |
|  | comp34222_c0 | XP_393895.2 | 1.14E-78 | PREDICTED: ras-related protein M-Ras-like [Apis mellifera] |
|  | comp36143_c0 | XP_972376.2 | 1.94E-37 | PREDICTED: similar to MRAS2, putative [Tribolium castaneum] |
|  | comp43215_c0 | XP_972376.2 | 1.60E-32 | PREDICTED: similar to MRAS2, putative [Tribolium castaneum] |
| TRKA | comp15050_c0 | AAX94284.1 | 3.69E-81 | neurotrophic tyrosine kinase receptor precursor [Branchiostoma floridae] |
| G12 | comp19342_c0 | EFX86199.1 | 8.35E-18 | guanine nucleotide binding protein, gamma subunit [Daphnia pulex] |
|  | comp53422_c0 | EFX86199.1 | 8.35E-18 | guanine nucleotide binding protein, gamma subunit [Daphnia pulex] |
|  | comp360507_c0 | EFX86199.1 | 8.35E-18 | guanine nucleotide binding protein, gamma subunit [Daphnia pulex] |
|  | comp21435_c1 | EGI64184.1 | 3.12E-133 | Guanine nucleotide-binding protein subunit alpha-like protein [Acromyrmex echinatior] |
|  | comp240505_c0 | EGI64184.1 | 3.12E-133 | Guanine nucleotide-binding protein subunit alpha-like protein [Acromyrmex echinatior] |
|  | comp555432_c0 | EGI64184.1 | 3.12E-133 | Guanine nucleotide-binding protein subunit alpha-like protein [Acromyrmex echinatior] |
| Gap1m | comp42268_c0 | XP_001945701.2 | 0 | PREDICTED: probable Ras GTPase-activating protein-like [Acyrthosiphon pisum] |
|  | comp44183_c0 | XP_001945701.2 | 2.60E-111 | PREDICTED: probable Ras GTPase-activating protein-like [Acyrthosiphon pisum] |
| p120GAP | comp17195_c0 | XP_001942745.1 | 0 | PREDICTED: ras GTPase-activating protein 1-like [Acyrthosiphon pisum] |
|  | comp41773_c0 | XP_001942745.1 | 0 | PREDICTED: ras GTPase-activating protein 1-like [Acyrthosiphon pisum] |
| NF1 | comp45632_c0 | XP_003402236.1 | 3.29E-64 | PREDICTED: neurofibromin-like [Bombus terrestris] |
|  | comp45632_c1 | XP_001602698.1 | 0 | PREDICTED: similar to neurofibromin [Nasonia vitripennis] |
| CNrasGEF | comp45641_c0 | XP_001952587.1 | 0 | PREDICTED: rap guanine nucleotide exchange factor 2-like [Acyrthosiphon pisum] |
|  | comp33052_c0 | XP_002732773.1 | 3.29E-28 | PREDICTED: Rap guanine nucleotide exchange factor 2-like [Saccoglossus kowalevskii] |
|  | comp137682_c0 | XP_002732773.1 | 3.29E-28 | PREDICTED: Rap guanine nucleotide exchange factor 2-like [Saccoglossus kowalevskii] |
| PKA | comp17094_c0 | XP_002423307.1 | 2.91E-155 | cAMP-dependent protein kinase catalytic subunit, putative [Pediculus humanus corporis] |
|  | comp234788_c0 | XP_002423307.1 | 2.91E-155 | cAMP-dependent protein kinase catalytic subunit, putative [Pediculus humanus corporis] |
|  | comp43718_c5 | XP_973065.1 | 3.14E-133 | PREDICTED: similar to camp-dependent protein kinase catalytic subunit [Tribolium castaneum] |
| PKC | comp21023_c0 | XP_002410223.1 | 5.10E-48 | protein kinase C, putative [Ixodes scapularis] |
|  | comp26562_c0 | XP_002410223.1 | 5.10E-48 | protein kinase C, putative [Ixodes scapularis] |
|  | comp33888_c0 | XP_001601074.1 | 0 | PREDICTED: similar to conventional protein kinase C [Nasonia vitripennis] |
|  | comp124627_c0 | XP_001601074.1 | 1.45E-34 | PREDICTED: similar to conventional protein kinase C [Nasonia vitripennis] |
| Rap1 | comp39006_c0 | ACJ66625.1 | 7.77E-90 | Ras protein [Fenneropenaeus chinensis] |
|  | comp39006_c1 | ACJ66625.1 | 7.77E-90 | Ras protein [Fenneropenaeus chinensis] |
| IKK | comp37279_c0 | XP_001927565.2 | 5.60E-56 | I-kappa-B kinase [Crassostrea gigas] |
|  | comp150341_c0 | XP_001927565.2 | 3.02E-47 | PREDICTED: tonsoku-like protein-like isoform 1 [Sus scrofa] |
|  | comp162911_c0 | AAC05683.1 | 3.02E-47 | PREDICTED: tonsoku-like protein-like isoform 1 [Sus scrofa] |
| NF-κB | comp43492_c1 | ADM14334.1 | 4.60E-115 | relish [Eriocheir sinensis] |
|  | comp43492_c2 | ADM14334.1 | 0 | relish [Eriocheir sinensis] |
|  | comp43492_c3 | ADM14334.1 | 1.03E-168 | relish [Eriocheir sinensis] |
| ERK | comp36917_c0 | ACX32460.1 | 0 | mitogen-activated protein kinase [Scylla paramamosain] |
|  | comp39561_c1 | NP_001036922.1 | 1.90E-158 | MAP kinse-ERK kinase [Bombyx mori] |
| PTP | comp23050_c0 | EFN69088.1 | 3.56E-60 | Receptor-type tyrosine-protein phosphatase R [Camponotus floridanus] |
|  | comp27866_c0 | XP_002428180.1 | 2.04E-14 | tyrosine-protein phosphatase non-receptor type, putative [Pediculus humanus corporis] |
| MKP | comp36734_c1 | XP_002430571.1 | 4.24E-115 | dual specificity protein phosphatase, putative [Pediculus humanus corporis] |
|  | comp40902_c0 | ACO12527.1 | 2.33E-35 | Dual specificity protein phosphatase 3 [Lepeophtheirus salmonis] |
|  | comp227779_c0 | XP_001945515.2 | 9.10E-47 | PREDICTED: dual specificity protein phosphatase 12-like [Acyrthosiphon pisum] |
|  | comp427030_c0 | XP_001945515.2 | 9.10E-47 | PREDICTED: dual specificity protein phosphatase 12-like [Acyrthosiphon pisum] |
| TAU | comp42288_c0 | XP_001955318.1 | 1.60E-56 | GF18699 [Drosophila ananassae] |
| STMN | comp42386_c1 | EGI59233.1 | 8.12E-49 | Stathmin-4 [Acromyrmex echinatior] |
| cPLA2 | comp39689_c0 | XP_002127884.1 | 9.58E-53 | PREDICTED: similar to Cytosolic phospholipase A2 (cPLA2) (Phospholipase A2 group IVA) [Ciona intestinalis] |
|  | comp267438_c0 | XP_002127884.1 | 9.58E-53 | PREDICTED: similar to Cytosolic phospholipase A2 (cPLA2) (Phospholipase A2 group IVA) [Ciona intestinalis] |
| RSK2 | comp43663_c1 | XP_002432758.1 | 9.42E-24 | Ribosomal protein S6 kinase alpha-2, putative [Pediculus humanus corporis] |
| Elk-1 | comp39691_c0 | XP_002429096.1 | 8.37E-67 | protein C-ets-1-B, putative [Pediculus humanus corporis] |
| Sapla | comp37140_c0 | XP_002410379.1 | 2.39E-47 | ETS domain-containing protein Elk-4, putative [Ixodes scapularis] |
| Myc | comp42818_c4 | EFX79343.1 | 9.02E-16 | Myc, dMyc-like protein [Daphnia pulex] |
| SRF | comp24104_c0 | CAB62047.1 | 5.35E-51 | Serum Response Factor [Artemia franciscana] |
| PPP3C | comp33500_c0 | ADD19580.1 | 1.35E-85 | Ca2+/calmodulin-dependent protein phosphatase [Glossina morsitans morsitans] |
|  | comp42122_c0 | XP_001602102.1 | 0 | PREDICTED: similar to calcineurin A [Nasonia vitripennis] |
| FASL | comp44048_c0 | AEK86525.1 | 3.86E-80 | TNFSF [Litopenaeus vannamei] |
|  | comp584797_c0 | AEK86525.1 | 3.86E-80 | TNFSF [Litopenaeus vannamei] |
| FAS | comp36962_c0 | AEK86527.1 | 8.05E-34 | TNFRSF [Litopenaeus vannamei] |
| TGFBR | comp28860_c0 | XP_002412676.1 | 2.15E-160 | transforming growth factor-beta receptor type I, putative [Ixodes scapularis] |
| TRAF6 | comp13680_c0 | ADM26237.1 | 2.45E-21 | tumor necrosis factor receptor-associated factor 6 [Litopenaeus vannamei] |
|  | comp40743_c0 | XP_002426151.1 | 3.07E-60 | TNF receptor-associated factor, putative [Pediculus humanus corporis] |
| CASP | comp34703_c0 | XP_003385047.1 | 1.33E-20 | PREDICTED: caspase-3-like [Amphimedon queenslandica] |
|  | comp466192_c0 | ADM45311.1 | 6.08E-32 | caspase [Eriocheir sinensis] |
|  | comp587122_c0 | ADM45311.1 | 6.08E-32 | caspase [Eriocheir sinensis] |
| DAXX | comp29315_c0 | XP_002735579.1 | 9.16E-29 | PREDICTED: death-domain associated protein-like [Saccoglossus kowalevskii] |
|  | comp29812_c0 | XP_002735579.1 | 9.16E-29 | PREDICTED: death-domain associated protein-like [Saccoglossus kowalevskii] |
|  | comp29812_c1 | XP_002735579.1 | 9.16E-29 | PREDICTED: death-domain associated protein-like [Saccoglossus kowalevskii] |
|  | comp37820_c0 | AAY56093.1 | 8.30E-14 | death-associated protein-like [Penaeus monodon] |
| ECSIT | comp42144_c1 | BAI40012.1 | 4.42E-114 | evolutionarily conserved signaling intermediate in Toll pathways [Marsupenaeus japonicus] |
| PP2CB | comp42525_c0 | NP_001008030.1 | 1.21E-134 | protein phosphatase, Mg2+/Mn2+ dependent, 1B [Xenopus (Silurana) tropicalis] |
| cdc42/Rac | comp39531_c0 | XP_003393447.1 | 3.38E-100 | PREDICTED: cdc42 homolog [Bombus terrestris] |
|  | comp42743_c4 | XP_001660307.1 | 1.27E-98 | rac gtpase [Aedes aegypti] |
|  | comp103709_c0 | XP_002428346.1 | 3.00E-98 | RAC GTPase, putative [Pediculus humanus corporis] |
| PAK1/2 | comp13148_c0 | XP_002131099.1 | 6.96E-127 | PREDICTED: similar to p21 (CDKN1A)-activated kinase 1 [Ciona intestinalis] |
|  | comp35813_c0 | XP_002426989.1 | 1.33E-139 | CDC42 GTPase-activating protein, putative [Pediculus humanus corporis] |
|  | comp39666_c0 | XP_003251334.1 | 0 | PREDICTED: serine/threonine-protein kinase PAK 1 isoform 2 [Apis mellifera] |
|  | comp39905_c2 | EGI64863.1 | 6.98E-114 | Serine/threonine-protein kinase PAK 1 [Acromyrmex echinatior] |
| MST1/2 | comp13995_c0 | EGI57844.1 | 1.02E-157 | Serine/threonine-protein kinase 3 [Acromyrmex echinatior] |
|  | comp30260_c1 | EGI57844.1 | 1.02E-157 | Serine/threonine-protein kinase 3 [Acromyrmex echinatior] |
|  | comp41120_c1 | XP_002433209.1 | 3.85E-26 | serine/threonine-protein kinase, putative [Pediculus humanus corporis] |
| MEKK1 | comp35325_c1 | XP_424734.2 | 1.27E-44 | PREDICTED: similar to MEK kinase 1 [*Gallus gallus*] |
|  | comp42183_c1 | XP_002408296.1 | 1.02E-21 | mitogen activated protein kinase kinase kinase 1, MAPKKK1, MEKK1, putative [*Ixodes scapularis*] |
| LZK | comp42917_c0 | XP_003396640.1 | 1.42E-164 | PREDICTED: mitogen-activated protein kinase kinase kinase 13-like isoform 2 [Bombus terrestris] |
| TAO | comp37742_c0 | XP_002426013.1 | 4.18E-124 | predicted protein [Pediculus humanus corporis] |
| FLNA | comp42005_c2 | EFX70014.1 | 2.10E-12 | hypothetical protein DAPPUDRAFT_328543 [Daphnia pulex] |
| JIP3 | comp30872_c0 | XP_003395970.1 | 0 | PREDICTED: LOW QUALITY PROTEIN: JNK-interacting protein 3-like [Bombus terrestris] |
|  | comp42257_c0 | XP_003395970.1 | 0 | PREDICTED: LOW QUALITY PROTEIN: JNK-interacting protein 3-like [Bombus terrestris] |
|  | comp44565_c2 | XM_003354659.1 | 5.17E-37 | PREDICTED: Sus scrofa mitogen-activated protein kinase 8 interacting protein 3, transcript variant 2 (MAPK8IP3) |
| HSP72 | comp11344_c0 | XP_001827946.1 | 5.56E-161 | molecular chaperone [Enterocytozoon bieneusi H348] |
|  | comp30331_c0 | CAL68989.1 | 3.04E-101 | heat shock protein 70 kDa [Cyanagraea praedator] |
|  | comp38443_c0 | CAL68995.1 | 2.27E-117 | heat shock protein 70 kDa [Cancer pagurus] |
|  | comp149772_c0 | ACZ02405.1 | 7.64E-31 | heat shock protein 70 [Portunus trituberculatus] |
|  | comp314066_c0 | ACF98297.1 | 0 | heat shock protein 70 [Eriocheir sinensis] |
| ARRB | comp45471_c3 | EFX68939.1 | 0 | hypothetical protein DAPPUDRAFT_329620 [Daphnia pulex] |
| Crk | comp42693_c0 | XP_002427598.1 | 5.89E-106 | Adapter molecule Crk, putative [Pediculus humanus corporis] |
| MKK4 | comp38478_c0 | EFN81517.1 | 4.92E-152 | Dual specificity mitogen-activated protein kinase kinase 4 [Harpegnathos saltator] |
| JIP1/2 | comp36172_c0 | XP_392444.3 | 1.23E-115 | PREDICTED: JNK-interacting protein 1 [Apis mellifera] |
| JNK | comp35816_c1 | BAI87826.1 | 0 | c-jun N-terminal kinase [Marsupenaeus japonicus] |
| JUN | comp44267_c0 | EGI68820.1 | 1.90E-49 | Transcription factor AP-1 [Acromyrmex echinatior] |
| AKT | comp43162_c2 | ADM87425.3 | 0 | Akt [Gecarcinus lateralis] |
| PP5 | comp44603_c0 | XP_971407.1 | 0 | PREDICTED: similar to protein phosphatase-5 [Tribolium castaneum] |
| P38 | comp34662_c0 | ADT91683.1 | 8.07E-169 | p38 mitogen-activated protein kinase [Apis cerana cerana] |
| p53 | comp44289_c7 | XP_968601.2 | 2.59E-102 | PREDICTED: similar to apoptosis stimulating of P53 [Tribolium castaneum] |
|  | comp45072_c0 | ACQ58385.1 | 2.54E-13 | p53 and DNA damage-regulated protein 1 [Anoplopoma fimbria] |
| ATF2 | comp44533_c0 | XP_001515843.1 | 1.13E-16 | PREDICTED: similar to activating transcription factor 2 [Ornithorhynchus anatinus] |
| MAX | comp37974_c0 | XP_003401810.1 | 7.59E-38 | PREDICTED: protein max-like isoform 1 [Bombus terrestris] |
| MEF2C | comp35942_c0 | XP_971771.1 | 1.56E-82 | PREDICTED: similar to myocyte-specific enhancer factor 2d [Tribolium castaneum] |
| MSK1/2 | comp20786_c2 | XP_002431024.1 | 3.20E-21 | Ribosomal protein S6 kinase alpha-5, putative [Pediculus humanus corporis] |
|  | comp34323_c0 | XP_002431024.1 | 3.20E-21 | Ribosomal protein S6 kinase alpha-5, putative [Pediculus humanus corporis] |
| MAPKAPK | comp23135_c0 | ABC25082.1 | 7.31E-87 | MAP kinase activated protein-kinase-2 [Glossina morsitans morsitans] |
|  | comp45432_c1 | ABC25082.1 | 7.31E-87 | MAP kinase activated protein-kinase-2 [Glossina morsitans morsitans] |
